# A Pre-Exposure Prophylaxis Adherence Intervention (LifeSteps) for Young Men Who Have Sex With Men: Protocol for a Pilot Randomized Controlled Trial

**DOI:** 10.2196/10661

**Published:** 2019-01-29

**Authors:** Katie B Biello, Christina Psaros, Douglas S Krakower, Elliot Marrow, Steven A Safren, Matthew J Mimiaga, Lisa Hightow-Weidman, Patrick Sullivan, Kenneth H Mayer

**Affiliations:** 1 Center for Health Equity Research Brown University School of Public Health Providence, RI United States; 2 Department of Behavioral & Social Sciences Brown University School of Public Health Providence, RI United States; 3 The Fenway Institute Fenway Health Boston, MA United States; 4 Department of Epidemiology Brown University School of Public Health Providence, RI United States; 5 Department of Psychiatry Massachusetts General Hospital Boston, MA United States; 6 Department of Psychology Harvard Medical School Boston, MA United States; 7 Department of Medicine Harvard Medical School Boston, MA United States; 8 Division of Infectious Diseases Beth Israel Deaconess Medical Center Boston, MA United States; 9 Department of Psychology University of Miami Coral Gables, FL United States; 10 Behavior and Technology Lab Institute for Global Health and Infectious Diseases University of North Carolina at Chapel Hill Chapel Hill, NC United States; 11 Department of Epidemiology Rollins School of Public Health Emory University Atlanta, GA United States; 12 Department of Global Health and Population Harvard TH Chan School of Public Health Boston, MA United States

**Keywords:** adolescents, adherence, antiretroviral pre-exposure prophylaxis, HIV prevention, men who have sex with men

## Abstract

**Background:**

New HIV infections occur at a disproportionately high rate among young men who have sex with men (YMSM). It is, therefore, essential that comprehensive HIV prevention strategies, specifically tailored to their needs and perceptions, are developed, tested, and disseminated. Antiretroviral pre-exposure prophylaxis (PrEP) is effective in decreasing HIV transmission among men who have sex with men; however, adherence is critical to its efficacy. In open-label studies among YMSM, adherence was suboptimal. Hence, behavioral approaches that address the unique challenges to YMSM PrEP adherence are needed.

**Objective:**

This study aims to describe the protocol for intervention refinement and a pilot randomized controlled trial (RCT) of a PrEP adherence intervention, LifeSteps for pre-exposure prophylaxis for young men who have sex with men (LSPY).

**Methods:**

This study includes the following 2 phases: formative qualitative interviews with approximately 20 YMSM and 10 key informants for intervention adaptation and refinement and a pilot RCT of up to 50 YMSM to assess the feasibility, acceptability, and preliminary efficacy of the LSPY, compared with the PrEP standard of care, to improve PrEP adherence. Participants will be recruited at 3 iTech subject recruitment venues in the United States.

**Results:**

Phase 1 is expected to begin in June 2018, and enrollment of phase 2 is anticipated to begin in early 2019.

**Conclusions:**

Few rigorously developed and tested interventions have been designed to increase PrEP adherence among YMSM in community settings, despite this population’s high HIV incidence. The long-term goal of this intervention is to develop scalable protocols to optimize at-risk YMSM’s PrEP uptake and adherence to decrease the HIV incidence.

**International Registered Report Identifier (IRRID):**

DERR1-10.2196/10661

## Introduction

### Background

In the United States, men who have sex with men (MSM) represent over half of all individuals living with HIV (56%) [1,2] and account for the largest number of new HIV infections each year (70%), with rates of new diagnoses at least 44 times higher than rates among heterosexual men [1,2]. While the incidence of new infections has decreased among other groups (eg, heterosexuals and injection drug users), the annual number of new infections among MSM has consistently increased over the past 20 years [1]. New HIV infections occur at a disproportionately high rate among young men who have sex with men (YMSM) in particular [3,4]. As such, it is essential that comprehensive HIV prevention strategies, specifically tailored to the needs and perceptions of YMSM, are developed, tested, and disseminated.

Pre-exposure prophylaxis (PrEP) is currently the only Food and Drug Administration-approved biomedical prevention method for MSM in the United States. The iPrEx study [5], which recruited 2499 men and transgender women over 11 sites in 6 countries, represented the first proof-of-concept that oral chemoprophylaxis is effective in decreasing the HIV transmission among MSM. However, adherence is critical to PrEP efficacy. In the iPrEx study, among participants with detectable levels of tenofovir in their blood, the risk of acquiring HIV decreased by >90%, and the intent-to-treat efficacy was 86% in 2 subsequent TDF/FTC (emtricitabine/tenofovir disoproxil fumarate, brand name Truvada) PrEP clinical trials of MSM in the United Kingdom and France [6,7]. In addition, pharmacological analyses corroborated the highly protective effects of TDF/FTC for PrEP among individuals who had detectable medication levels in their blood, highlighting the critical role of adherence in PrEP efficacy. This underscores the need for future PrEP interventions to focus on evidence-based strategies to promote adherence, to optimize the benefits that antiretroviral chemoprophylaxis may be able to provide for at-risk MSM.

Although YMSM readily accepted PrEP in several studies, adherence was suboptimal. In an open-label study of PrEP use by YMSM aged 18-22 years (ATN 110), only one-third of the study participants had protective drug levels after 1 year despite intensive adherence counseling, and the HIV incidence in the sample was 3% per year [8]. In addition, adherence was suboptimal in a parallel PrEP study of YMSM aged 15-17 years, and the annualized HIV incidence exceeded 6% [9]. In these studies, adherence tended to decline after 3 months of PrEP use, when the interval between study visits was extended from monthly to every 3 months, despite individualized or group-based behavioral adherence interventions. PrEP adherence might be even lower for YMSM prescribed PrEP in primary care settings, where adherence support may be less intensive than that in clinical trials. Thus, tailored interventions to support PrEP adherence in YMSM are needed.

Interventions to support PrEP adherence have shown promise in adults, but require adaptation to meet the unique needs of adolescents. LifeSteps is an evidence-based HIV medication adherence intervention for HIV-infected individuals, which was developed by Safren et al [10-12]; it has been adapted for diverse populations [13-15], including adolescents in the “Positive STEPS” project [16]. Positive STEPS was successful in improving antiretroviral therapy adherence relative to a standard-of-care comparison group in a pilot randomized controlled trial (RCT) among HIV-infected youth, aged 16-24 years, in the United States and is currently being evaluated in a National Institutes of Health–funded, 2-city efficacy trial (NCT03092531). In addition, LifeSteps has been adapted for PrEP users. In a study of at-risk MSM aged ≥18 years, a 4-session, nurse-delivered version of LifeSteps adapted for PrEP users resulted in excellent PrEP adherence and higher drug levels in the intervention condition compared with a time- and attention-matched control condition [17]. Given the evidence that shows short message service (SMS) text messages can improve antiretroviral medication adherence when integrated with counseling [18-23], especially in adolescents, the use of weekly SMS text messages as motivational and social cues to support adherence was added to LifeSteps [17,24].

Many YMSM may be dealing with a variety of unique psychosocial (eg, sexual identity formation, depression, and substance use) and sociostructural (eg, stigma, bullying, unstable housing, and family trauma) concerns, creating potential barriers to PrEP adherence that require additional support, for them to optimally adhere to and achieve maximal benefit from PrEP. To determine the extent to which these factors might influence PrEP adherence, LifeSteps for PrEP is being further optimized for YMSM through formative interviews with at-risk YMSM and their providers, and with a subsequent pilot study.

### Theoretical Framework for Intervention

The adaptation of LifeSteps for PrEP for YMSM (LSPY) is being guided by the Gelberg-Andersen Behavioral Model for Vulnerable Populations; this model posits that health behaviors are influenced by a complex interplay of *environmental* and *patient* factors [25]. For at-risk YMSM, *environmental factors* that may affect adherence include challenges they face in their *external environments* (eg, unstable housing) and those faced in *health care environments* (eg, relying on parents’ insurance) [26]. *Patient*, or *individual*, factors that affect PrEP adherence include *predisposing factors* (eg, low health literacy), *enabling factors* (eg, copay assistance), and *perception of need* (eg, HIV risk perception) [27]. The proposed intervention—through the LifeSteps for PrEP modules and daily SMS text messages—aims to exert its effects on multiple domains of the model to optimize PrEP adherence.

### Aims and Objectives

The long-term goal is to develop scalable protocols to optimize at-risk YMSM’s PrEP uptake and adherence to decrease the HIV incidence. The first step toward this goal is to revise and refine LifeSteps for PrEP for delivery by nurses specializing in adolescent health so that it is tailored for high-risk, HIV-uninfected YMSM initiating PrEP. This paper aims to describe the protocol for the refinement of LSPY and a pilot RCT to examine the acceptability and feasibility of LSPY. We hypothesize that participants who are randomized to LifeSteps for PrEP will be highly satisfied with the intervention. In addition, we hypothesize that, although not powered to detect significant differences, YMSM randomized to the LifeSteps for PrEP condition will demonstrate better adherence compared with YMSM in the standard-of-care condition.

## Methods

### Phase 1: LifeSteps for Pre-Exposure Prophylaxis for Young Men Who Have Sex With Men Refinement

To refine the LSPY intervention, we will conduct in-depth, individual qualitative interviews with up to 20 HIV-uninfected, at-risk YMSM who present for bacterial sexually transmitted infection (STI) screening or treatment, or those seeking PrEP at Fenway Health, an iTech subject recruitment venue (SRV) and clinical center, which specializes in the care of sexual and gender minority patients [28]. In addition, we will conduct in-depth qualitative interviews with up to 10 key informants, including PrEP providers and staff, at community-based organizations that work with YMSM. Youth participants will be HIV-uninfected YMSM aged 15-24 years who self-report evidence of high risk for acquiring HIV infection (eg, recent bacterial STI diagnosis). We will use purposive sampling to recruit a diverse sample of YMSM with respect to race and ethnicity, age, and prior PrEP experience. Furthermore, we will recruit YMSM at various points in the PrEP continuum of care, including those who have opted not to initiate PrEP despite recommendations from clinicians, those who are using PrEP and report high levels of adherence, and those who report adherence challenges.

After informed consent and prior to the interview, participants will complete a brief demographic and behavioral questionnaire to contextualize the qualitative data. In the interviews, we will identify potential strategies to optimize PrEP adherence for YMSM and will explore youth perspectives on the use of nurses to deliver the intervention and weekly SMS text messages.

For PrEP-naïve youth, we will also explore youth concerns about adhering to PrEP, how these concerns influence their decisions about whether or not to initiate PrEP, and whether the availability of a structured, supportive intervention that was nurse-delivered or regular SMS text messages would influence these decisions.

For PrEP-experienced youth, we will explore their experiences with medication adherence and strategies used to overcome any barriers, their views on the content of what they might have wanted to discuss with a nurse (eg, potential side effects, lab monitoring, how to discuss PrEP with friends and family, and substance use), and acceptability of SMS text messaging.

Specific topics to be explored are based on the conceptual model described above, including: environmental factors affecting access and PrEP adherence, including structural factors (eg, housing insecurity and lack of transportation), health care factors (eg, inconvenient scheduling of clinical visits and nonaffirming atmosphere for sexual minorities); patient factors that may influence adherence, including predisposing issues (eg, substance use and depression), enabling factors (eg, knowledge of the benefits of PrEP and social supports), and perceived need to use PrEP (eg, self-perception of HIV risk); and perspectives regarding the proposed adherence interventions, including the use of SMS text messaging as reminders and motivational cues (eg, ideal timing and content), and a nurse-led intervention (eg, reasons they would or would not want to discuss adherence with a nurse, the preferred content, and structure of counseling sessions). An open-ended approach will allow us to elicit potentially unexpected considerations that influence initiation of and PrEP adherence among YMSM.

Study visits will last approximately 60-90 minutes, and participants will receive US $50 for this one-time interview. All interviews will be digitally recorded, and interviewers will take detailed notes using debriefing forms. Recordings will be transcribed by members of the iTech Analytic Core who are trained in qualitative methods. The qualitative team will apply rapid qualitative analysis techniques to the analysis of interview data [29]; this approach involves the initial identification of themes and tabulation of frequencies regarding the endorsement of themes across participants. Immediately following each of the interviews, the facilitator team will record observational insights, content, and key themes from the interview; this approach will result in an ongoing set of memos created by the team that rapidly describes and elucidates salient themes. The memos will guide the codebook creation and coding scheme for a more formal content analysis of transcripts [30,31]. These data will inform the adaptation of the youth-tailored LSPY intervention that we will test in the RCT pilot. Specifically, new modules may be developed, other modules removed, or new content added to existing modules. If diverse needs are identified by phase 1 participants, refinements will be made globally given that the intervention is designed to be individually tailored by the interventionists during the sessions based on participants’ needs and clinical judgment [32].

### Phase 2: Pilot Randomized Controlled Trial

#### Trial Registration, Ethics, Consent, and Institutional Board Approval

The research and ethics presented in this study have been reviewed and approved by the University of North Carolina at Chapel Hill Institutional Review Board (17-2513). A Certificate of Confidentiality has been obtained from the National Institute of Child Health and Human Development, and a waiver of parental consent will be obtained for participants aged 15-17 years. Furthermore, the study is in the process of being registered on ClinicalTrials.gov.

All participants will undergo screening in a private room at the clinical research site. If eligible (see below), informed consent will be conducted at this time. The informed consent documents will include detailed information on all study procedures and answer questions concerning the study and consent process.

#### Study Design

**Figure 1:**
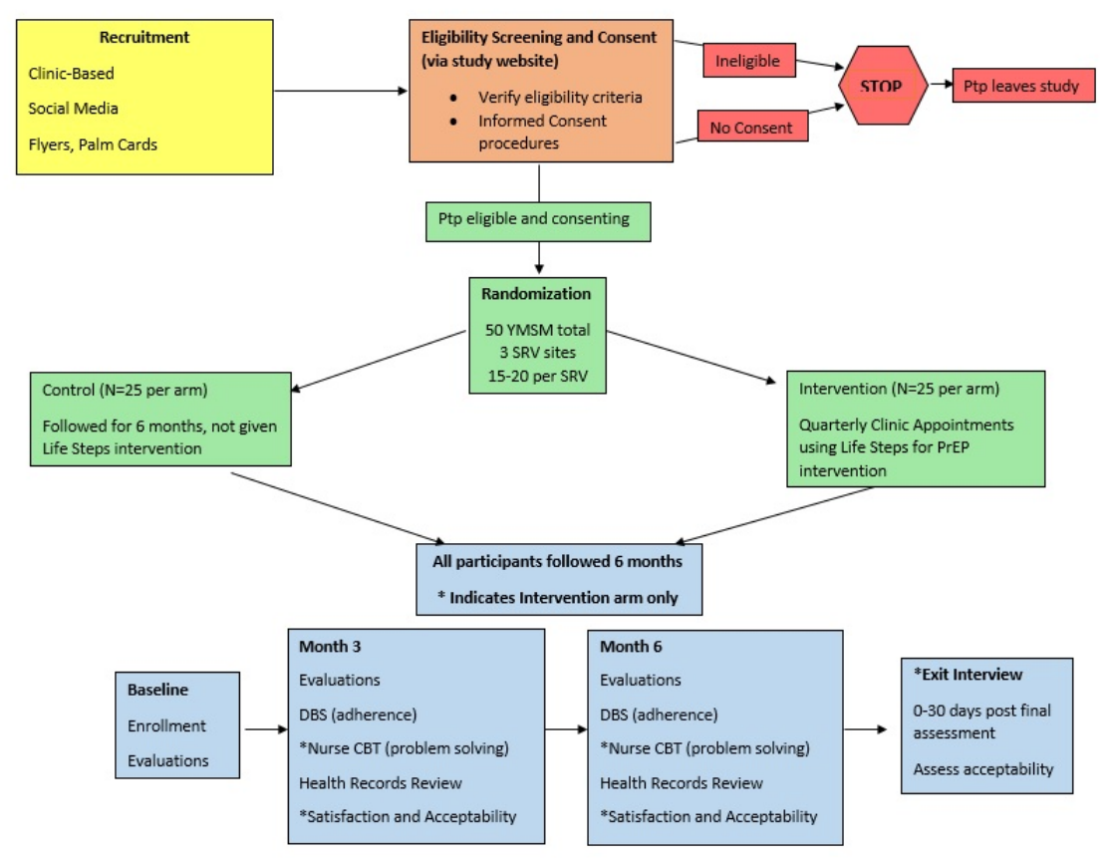
The pilot randomized controlled trial RCT study schema. Ptp: participant; YMSM: men who have sex with men; SRV: subject recruitment venue; PrEP: pre-exposure prophylaxis; DBS: dried blood spot; CBT: cognitive behavioral therapy.

A 3-site, 2-arm pilot RCT will be conducted to assess the feasibility and acceptability of the LSPY intervention and preliminary efficacy of the intervention to improve PrEP adherence for daily oral PrEP and retention in PrEP care, compared with a standard of PrEP care control group. The inclusion of a control group will allow us to estimate an effect size to sufficiently power a future full-scale efficacy trial. As such, we will enroll up to 50 YMSM in the RCT (randomized 1:1 to the 2 arms) from iTech SRVs in Atlanta (GA), Boston (MA), and Chicago (IL). Participants will be followed for 6 months and will complete biological (ie, renal safety, STI screening, and drug levels) and behavioral assessments every 3 months, including self-reported PrEP adherence, sexual behaviors, and psychosocial health. In addition, we will conduct a brief, 15-minute semistructured exit interview with participants in the LSPY intervention arm to provide an opportunity for more in-depth and open-ended feedback on the intervention satisfaction and acceptability. These data will be used to finalize the intervention manual to enhance participant acceptability. Figure 1 shows the pilot RCT schema.

#### Participants

Study participants (n=50) will be assessed for eligibility by completing a brief screener. The inclusion criteria include the following: age 15-24 years; assigned male sex at birth; identify as male; identified as PrEP candidates by local clinicians because of self-reported risk or presentation with a new bacterial STI; and PrEP-naïve (assessed by self-report). In addition, participants will be able to understand, read, and speak English. For the pilot RCT, we will recruit YMSM prior to their first PrEP prescription, as the interventions are designed to be administered at the time of PrEP initiation.

#### Recruitment

Active recruitment will be carried out by the study staff at each SRV by recruiting men at the SRVs. In each city, the SRVs represent diverse settings, including an academic research center (Atlanta, GA), a community health center (Boston, MA), and a hospital-based clinic (Chicago, IL). In addition, recruitment will occur at local organizations and venues that YMSM attend, including community-based organizations for lesbian, gay, bisexual, and transgender youth, Youth Pride events, etc.

At recruitment venues, trained staff will approach youth and offer them information about the study (either verbally or by offering them a business card or advertisement flyer), including brief descriptions of the study design and contact information (ie, study email and phone number).

In addition, passive approaches for recruitment will include posting study information through flyers, posters, and palm cards describing the study at these venues. Moreover, Web-based recruitment will be conducted by placing banner advertisements on popular Web-based social media outlets for YMSM (eg, Facebook, Grindr, etc).

#### Randomization

Only participants who express interest in LSPY to increase PrEP adherence, meet the eligibility criteria, and provide informed consent will be eligible for randomization. Overall, 50 YMSM who are PrEP-naïve and appropriate candidates for antiretroviral PrEP at the 3 iTech SRVs (15-20 per site) will be randomized 1:1. The randomization will be stratified by SRVs [33] and will be based on a pregenerated list created by iTech Analytic Core statisticians and accessed by a Web portal.

#### Incentives

Participants in the pilot RCT will receive US $50 compensation for the in-person screening or baseline assessment and US $50 compensation for each completed follow-up assessment and the exit interview.

#### Intervention

##### Standard-of-Care Condition

Following the completion of baseline assessments, participants in both conditions will receive the standard of care for PrEP initiation and adherence. Each participating SRV will document standard-of-care procedures at their site prior to protocol initiation.

##### “LifeSteps For Pre-Exposure Prophylaxis For Young Men Who Have Sex With Men” Condition

The experimental intervention, LSPY, was derived from our prior work with individuals living with HIV [12,34] and individuals using PrEP [32], and will be finalized following phase 1 focus groups. LifeSteps was originally designed as a standalone, one-session adherence intervention for individuals living with HIV. The goal of the sessions was to help an individual understand all steps involved in successful adherence to HIV treatment (eg, communicating with the treatment team, medication storage, securing refills, etc). LifeSteps has since been refined for individuals using PrEP [17,32,35]. Phase 1 data may inform the structure of the sessions, the addition of optional modules, and problem-solving material tailored to the unique challenges of YMSM. Currently, the LSPY intervention consists of 4 weekly sessions at the time of PrEP initiation and 2 booster sessions, which occur 2 and 3 months after PrEP initiation. Overall, the core components of the intervention will focus on medication adherence, sexual behavior, and problem solving to overcome barriers to adherence, using motivational interviewing techniques when needed. Session 1 will include education about PrEP, a discussion involving the psychosocial context in which PrEP use occurs, a brief motivational interviewing exercise, and discussion of establishing a dosing schedule. The session content after session 1 is designed to be flexible, allowing patients to identify their adherence support needs and for the interventionist to choose the material that is most relevant to the individual participant. Session 2 will begin with an adherence “check-in,” and will, then, focus on understanding the clients’ experiences taking PrEP, and engaging in problem solving to address any reported barriers to adherence. In addition, session 3 will begin with an adherence check-in and will, then, introduce sexual risk behavior education, identifying high-risk activities, and factors that could increase and decrease personal risk for HIV, as well as other STIs. The session will involve a discussion about biological factors associated with the HIV transmission (eg, partners’ level of infectiousness and measured by plasma HIV RNA), as well as other STIs, and will discuss ways to reduce their risk in the context of taking PrEP. In the final weekly session, a nurse-counselor will discuss PrEP adherence goals and prior session content, and patients’ plans for continued PrEP use upon the intervention completion. Optional modules will provide a framework to help interventionists work with participants experiencing substance abuse or mental health concerns, which were adversely impacting PrEP adherence. For example, if a participant or an interventionist identifies a mental health or substance use-related concern that impacts PrEP adherence in the participant’s sexual health promotion plan, the interventionist may use motivational interviewing skills to increase the willingness to discuss this issue as a barrier to adherence or problem-solving strategies for managing it. These optional modules will also include site-specific referral sources (on-site, if relevant) so that more intensive counseling around the barrier may be sought. In addition, an interventionist may introduce a brief relaxation exercise if anxiety is a prominent concern. Booster sessions at months 2 and 3 are designed to offer an opportunity for the trained study nurse to assess PrEP adherence in the absence of weekly support. Study nurses can use booster sessions to review PrEP adherence over a longer time span and address barriers to adherence using problem-solving skills learned during the earlier sessions. For participants who identified no challenges to adherence, the study nurse can use the booster session to review and refine the existing adherence plan and help them identify potential future barriers to adherence.

As part of the LSPY intervention, *weekly SMS text messaging* will be used to support adherence, as well as to understand participants’ patterns of behavior. Participants will receive weekly SMS text messages to motivate them to take their medications and assess whether or not they took their medication and had condomless sex. The weekly texts will continue throughout the follow-up period for each participant in the intervention group.

#### Data Collection

Baseline assessments will be conducted in-person, with follow-up assessments after 3 and 6 months. At each major assessment, participants will complete an assessment battery through a secure Web-based data entry system. As mentioned previously, participants in the LSPY intervention arm will receive a weekly, brief SMS text messaging-based survey to assess whether or not they took their medication and had condomless sex in the past week. By obtaining data on the weekly patterns of medication adherence and HIV risk, the study team will be able to assess whether changes in adherence were associated with the increased, unchanged, or decreased HIV risk.

##### Primary Outcome Measures

To measure the *acceptability* of the LSPY intervention, participants will self-report the degree to which they find the intervention appropriate and useful using Likert-type agreement scales on factors such as the intervention content, intervention length, and intervention delivery. We will use the System Usability Scale, a validated 10-item measure, which is scored from 0 to 100 [37]. A score of ≥50 indicates that the intervention is acceptable [38].

To assess the *feasibility*, we will track the number of potential participants we screen, the number of potential participants who meet the study inclusion criteria, the number of participants who meet the study criteria and then enroll, and the number of treatment and assessment sessions completed by all enrolled participants (across conditions). In addition, we will track the duration of assessments and reasons for declining enrollment and prematurely leaving the trial.

Although this pilot study is not powered to examine the efficacy of biological and behavioral outcomes, we will assess its impact on adherence to obtain an estimated effect size to power a future full-scale efficacy trial. As such, at each major assessment, dried blood spot drug levels of tenofovir diphosphate and emtricitabine triphosphate will serve as biological correlates of *adherence* [39]. In addition, self-reported PrEP adherence will also be assessed using timeline follow back [36]. Furthermore, we will obtain medical record release forms from participants to determine medical appointment adherence, measured as the proportion of scheduled clinic visits attended by each patient over their study observation period.

##### Secondary Outcome Measures

To assess the impact of the intervention on potential mediators of adherence, we will use scales developed from our initial study [17,32] with adults. Specifically, *readiness to use PrEP* will be assessed using a series of questions that ask how likely they are to use or continue using PrEP under a variety of circumstances. In addition, *behavioral skills for PrEP use* will be assessed with 12 items that ask how “hard” or “easy” it was for participants to implement a variety of skills, including discussing side effects with medical providers and remembering to take pills on time. The *PrEP taking self-efficacy* will be adapted from the HIV Treatment Adherence Self-Efficacy Scale [40], which assesses confidence to take medications in various situations. Finally, we will also assess individual (eg, sexual behavior and mental health) and environmental (eg, incarceration, stigma, and health care access) covariates that could impact adherence.

#### Statistical Analyses

We will use descriptive statistics to characterize the distribution of all study variables. The primary analysis will measure the feasibility of the intervention by the proportion of participants retained in the study at the end of the study period, and we will measure the acceptability by the percentage of participants who rate each intervention as acceptable on their final follow-up survey. Point estimates of ≥.50 for the feasibility and acceptability will be considered the minimum criteria for the acceptability and feasibility, consistent with standards used in similar behavioral health studies.

The primary biological outcome analysis will compare adherence (defined by the percentage of participants with dried blood spot drug levels >700 fmol/punch, a level correlated with high protection from HIV acquisition [36,41]) at the 3- and 6-month visits between the study arms and will be used to estimate the effect size for a future full-scale efficacy trial. In addition, group differences in the proportion of PrEP clinic appointments kept will be compared.

All analyses will use 2-tailed tests of significance with significance at alpha=.05. We will follow an intent-to-treat model [42], analyzing participants in the study arm to which they were assigned. We will examine the equivalence of random assignment to groups with regards to key baseline characteristics, including sociodemographics, prior HIV testing patterns, and sexual risk-related variables. If randomization does not work to balance these characteristics, we will assess whether baseline differences may account for differences in outcomes.

## Results

While qualitative interviews are anticipated to begin in June 2018, recruitment for the pilot RCT is anticipated to begin in early 2019, with the study follow-up complete in February 2020.

## Discussion

Oral PrEP has the potential to change the HIV prevention landscape and curtail the HIV epidemic dramatically. Adequate adherence levels can reduce HIV acquisition among MSM by >90% [5,6,41,43], and increasing the effective use of PrEP among YMSM—one of the highest-risk groups for new infections—is one of the leading priorities for HIV prevention. However, YMSM face multiple challenges in initiating and adhering to PrEP [44], and, in 2 open-label studies of PrEP use by YMSM aged 15-17 and 18-22 years (ATN 113 and 110) [8,9], adherence was suboptimal after 3 monthly visits, and the HIV incidence was high (6% and 3%, respectively).

At present, few rigorously developed and tested interventions exist for increasing PrEP adherence among YMSM in community settings. Our long-term goal is to develop scalable protocols to optimize the at-risk YMSM’s PrEP uptake and adherence to decrease the HIV incidence. In a pilot RCT, LifeSteps for PrEP—a 4-session, nurse-delivered cognitive behavioral therapy-based counseling intervention—improved PrEP adherence compared with a time- and attention-matched controls among at-risk MSM aged ≥18 years [17].

The adaptation for LSPY will be informed, developed, and refined through formative research that involves YMSM at all levels. HIV-uninfected YMSM will inform the intervention and curricular materials through formative qualitative interviews and exit interviews after participating in the pilot RCT. By incorporating information and feedback on the content of the intervention from YMSM, we will ensure content is tailored to their contextual realities in a manner that promotes PrEP adherence skills building and problem solving.

Anticipated limitations of this protocol include potential limits of generalizability of the results of the formative, qualitative interviews given that they will take place in one urban city. While preparing for the pilot RCT, we will present the intervention at iTech-supported Youth Community Advisory Boards at participating SRVs to obtain additional feedback from geographically diverse populations. Similarly, the pilot RCT may have limited generalizability outside the 3 urban settings and may need to be further adapted for settings where access to clinical care is less robust.

If LSPY demonstrates the acceptability and feasibility in this pilot RCT, we plan to test its efficacy in a full-scale, multicity, randomized controlled efficacy trial. If shown to be efficacious, this in-person, nurse-delivered counseling intervention with SMS text messaging for ongoing support will allow for a youth-informed, targeted PrEP adherence intervention for YMSM.

## Abbreviations

LSPY: LifeSteps for pre-exposure prophylaxis for young men who have sex with men
MSM: men who have sex with men
PrEP: pre-exposure prophylaxis
RCT: randomized controlled trial
SMS: short message service
SRV: subject recruitment venue
STI: sexually transmitted infection
YMSM: young men who have sex with men
